# Development of a LAMP assay using hybridization-based TaqMan-style probes (HyTaq) for simultaneous detection of wild-type and macrolide-resistant *Mycoplasma pneumoniae*

**DOI:** 10.1128/jcm.01291-25

**Published:** 2025-12-17

**Authors:** Eunji Lee, Woo Sang Jung, Chae Seung Lim, Woong Sik Jang

**Affiliations:** 1 BK21 Graduate Program, Department of Biomedical Sciences, College of Medicine, Korea University 36899, Seongbuk-gu, Seoul, Republic of Korea; 2 Research and Development Department, Biozentech Co., Ltd., Geumcheon-gu, Seoul, Republic of Korea; 3 Department of Laboratory Medicine, College of Medicine, Korea University Guro Hospital 36899, Guro-gu, Seoul, Republic of Korea; 4 Department of Emergency Medicine, College of Medicine, Korea University Guro Hospital 58934, Guro-gu, Seoul, Republic of Korea; Children's Hospital Los Angeles, Los Angeles, California, USA

**Keywords:** *Mycoplasma pneumoniae*, macrolide resistance, 23S rRNA mutations (A2063G, A2064G), loop-mediated isothermal amplification (LAMP), isothermal SNP detection, TaqMan probe

## Abstract

*Mycoplasma pneumoniae* is a major cause of community-acquired pneumonia and increasingly exhibits macrolide resistance due to the A2063G and A2064G mutations in the 23S rRNA gene. We developed a loop-mediated isothermal amplification (LAMP) assay with hybridization-based TaqMan-style probes (HyTaq) to simultaneously detect *M. pneumoniae* wild-type strains and macrolide resistance mutations (A2063G and A2064G) within a single reaction (hereafter referred to as the MP-R HyTaq-LAMP assay). Analytical performance was evaluated using plasmid standards, and clinical validation was conducted with 224 nasopharyngeal swab samples. The MP-R HyTaq-LAMP assay detected wild-type strains and the A2063G and A2064G mutations with a limit of detection of 1 × 10³ copies/μL. In clinical samples, the assay demonstrated 95.79% sensitivity for A2063G and 100% sensitivity for wild-type strains, along with 100% specificity against 103 non-infection samples. Additionally, no cross-reactivity was observed with 16 other common respiratory pathogens. The MP-R HyTaq-LAMP assay provides rapid, multiplex detection of *M. pneumoniae* and associated macrolide resistance mutations without thermal cycling, demonstrating its strong potential as a point-of-care diagnostic tool.

**IMPORTANCE:**

Macrolide-resistant *Mycoplasma pneumoniae* is a significant cause of treatment failure in community-acquired respiratory infections, particularly in children and adolescents. Early and accurate detection of resistance-associated mutations, such as A2063G and A2064G, is essential for guiding effective antibiotic therapy. In this study, we developed an MP-R HyTaq-based loop-mediated isothermal amplification assay capable of simultaneously detecting and discriminating wild-type strains and macrolide resistance mutations (A2063G and A2064G) in a single-tube, isothermal reaction. Its high specificity, rapid turnaround time, and minimal equipment requirements make this assay suitable for point-of-care testing in diverse clinical settings.

## INTRODUCTION

*Mycoplasma pneumoniae* (*M. pneumoniae*) is a common etiologic agent of community-acquired respiratory tract infections (CAP), particularly in children and young adults (1, 2). It accounts for approximately 10%–40% of all CAP cases and up to 70% in closed or semi-closed settings, such as schools and military camps (3). Clinically, *M. pneumoniae* infection presents a wide spectrum of respiratory manifestations, ranging from mild upper respiratory illness to atypical pneumonia. Extrapulmonary complications—including dermatologic, neurologic, and cardiovascular involvement—have also been reported, further complicating diagnosis and treatment (4, 5). An important emerging concern is the high prevalence of macrolide-resistant *Mycoplasma pneumoniae* (MRMP), particularly in East Asia, where resistance rates among pediatric populations have exceeded 80% (6, 7). The primary mechanism of resistance involves point mutations in domain V of the 23S rRNA gene, most commonly A2063G and A2064G, which reduce the binding affinity of macrolides to their ribosomal target (8). As macrolides are frequently used empirically in children, rapid detection of these mutations is essential for appropriate therapeutic decision-making (9).

Traditional diagnostic methods for *M. pneumoniae* include culture, serology, and nucleic acid amplification tests (10, 11). Culture, while highly specific, is time-consuming and technically demanding due to the organism’s slow growth. Serologic assays, including IgM and IgG detection, often lack the sensitivity and specificity required for early diagnosis and cannot reliably distinguish acute from past infection. Currently, real-time PCR is considered the gold standard for *M. pneumoniae* detection due to its sensitivity and specificity; however, it requires thermal cycling instruments, centralized laboratories, and skilled personnel, which limits its applicability in resource-constrained settings (12, 13). Loop-mediated isothermal amplification (LAMP) has emerged as an attractive alternative to PCR. LAMP amplifies nucleic acids under isothermal conditions (typically 60°C–65°C) using a strand-displacing DNA polymerase and multiple primers targeting distinct regions of the gene (14, 15). This allows for rapid amplification within 30–60 minutes, without the need for complex equipment. LAMP has shown excellent potential for point-of-care applications, especially in low-resource or decentralized healthcare settings.

To enable real-time and specific detection in LAMP, various probe-based strategies have been developed, including assimilation probes (16), molecular beacons (17), fluorescence of loop primer upon self-dequenching-LAMP (18), and more recently, TaqMan-based LAMP assays (19). All these approaches were designed to overcome the limitations associated with non-specific intercalating dye-based detection methods. Among them, TaqMan probes are dual-labeled oligonucleotides bearing a fluorophore at the 5′ end and a quencher at the 3′ end, typically generating fluorescence upon cleavage by the 5′→3′ exonuclease activity of Taq DNA polymerase (20). However, since standard LAMP reactions utilize Bst polymerase, which lacks exonuclease activity, direct application of TaqMan probes to LAMP has been considered incompatible (21). A recently reported TaqMan probe-based LAMP system also relied on the original cleavage-dependent fluorescence mechanism (22). This was achieved by employing Bst 5.0 polymerase, which possesses 5′→3′ exonuclease activity, thereby circumventing the enzymatic limitation through the use of a modified polymerase.

In this study, we demonstrate for the first time that a conventional dual-labeled TaqMan-style probe can generate fluorescence solely through hybridization with its complementary target sequence, without enzymatic cleavage. Although structurally identical to traditional TaqMan probes, this previously unrecognized hybridization-based fluorescence mechanism enables probe function under standard LAMP conditions using Bst polymerase, which lacks 5′→3′ exonuclease activity. We term this probe system “HyTaq,” short for hybridization-based TaqMan-style probe. To apply this principle to clinically relevant targets, we designed the MP-R HyTaq-based loop-mediated isothermal amplification (HyTaq-LAMP) assay, where “MP-R” refers to *M. pneumoniae* and associated macrolide resistance mutations. This assay is capable of simultaneously detecting *M. pneumoniae* and differentiating wild-type (WT) strains from macrolide-resistant genotypes (A2063G and A2064G) in a single reaction. The analytical sensitivity of the assay was evaluated using recombinant plasmids and clinical samples. Clinical validation was performed on 224 nasopharyngeal (NP) swab specimens collected from patients at Korea University Guro Hospital.

## MATERIALS AND METHODS

### Clinical samples and DNA extraction

For clinical validation of the MP-R HyTaq-LAMP assay, a total of 224 NP swab specimens were collected between August and September 2024 at Korea University Guro Hospital (Seoul, South Korea). The specimens included 103 samples from patients infected with *M. pneumoniae* and 121 from non-infected individuals. Infection with *M. pneumoniae* was initially confirmed using the Allplex PneumoBacter Assay (Seegene Inc., South Korea), and point mutations in the 23S rRNA gene were further verified by direct sequencing performed by Macrogen Inc. (Seoul, South Korea). For the cross-reactivity evaluation, 16 NP swab specimens containing various respiratory pathogens were tested. Bacterial and fungal species included *Bordetella pertussis*, *Candida albicans*, *Escherichia coli*, *Klebsiella pneumoniae*, *Pseudomonas aeruginosa*, *Staphylococcus aureus*, *Staphylococcus epidermidis*, *Streptococcus agalactiae*, *Streptococcus pneumoniae*, and *Streptococcus pyogenes*. Viral pathogens included human parainfluenza virus types 1–4, human rhinovirus, and respiratory syncytial virus (RSV). The presence of each pathogen was confirmed using either commercial multiplex PCR kits (Seegene Inc., South Korea) or reference PCR assays based on previously published primer sets (23–30). All cross-reactivity tests were performed in triplicate. Nucleic acids were extracted from all specimens using the PowerEXP 32 automated extraction system (KogeneBiotech, South Korea) according to the manufacturer’s protocol. Briefly, 200 µL of each sample was loaded into a 96-well extraction plate for automated processing. Extracted DNA samples were stored at −20°C until further analysis. This study was conducted in accordance with the Declaration of Helsinki and was approved by the Institutional Review Board of Korea University Guro Hospital (approval number: 2025GR0008).

### Principle of the HyTaq-LAMP assay

**Fig 1:**
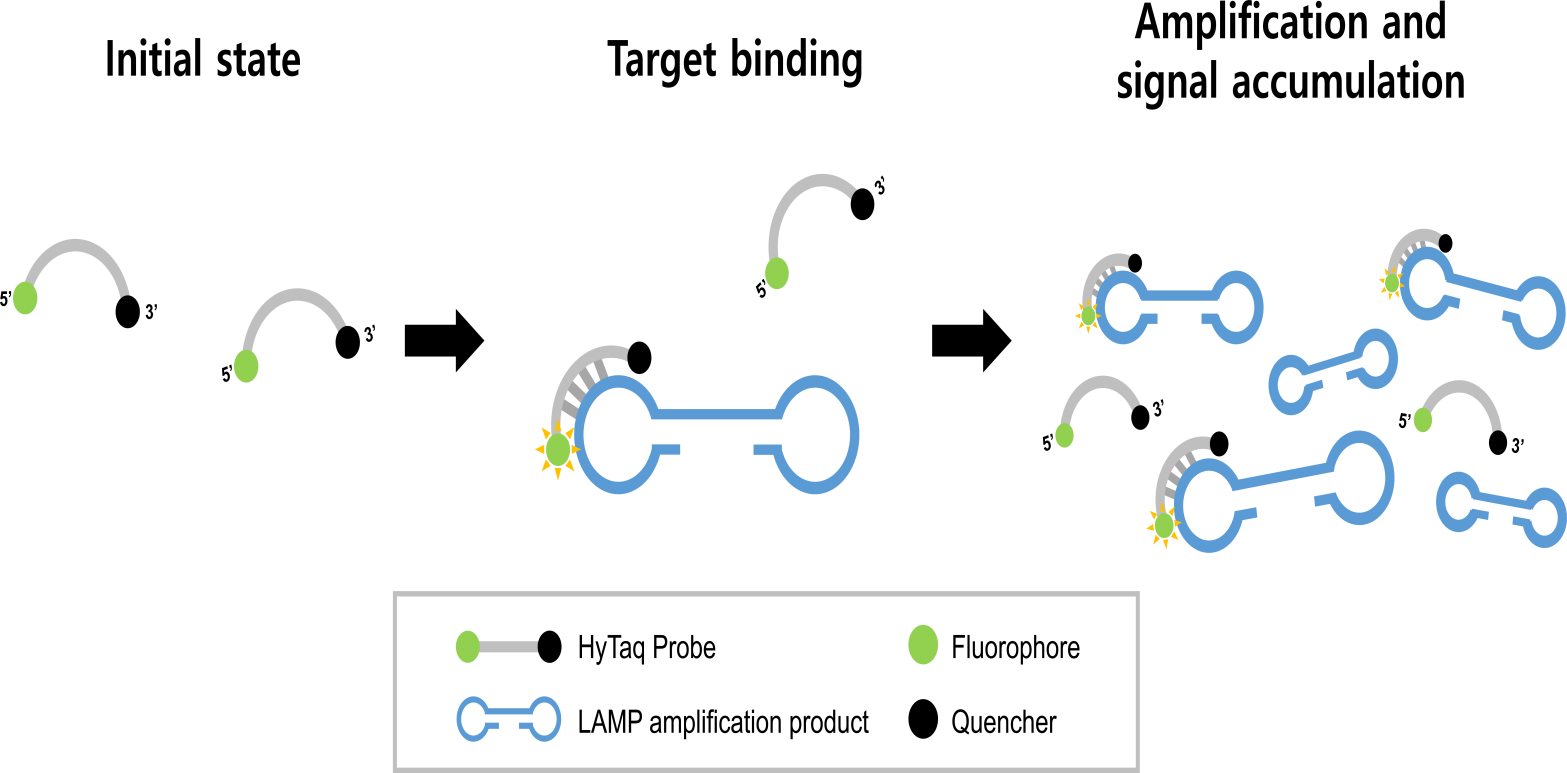
Schematic illustration of the HyTaq probe principle in LAMP. Fluorescence is restored upon hybridization to the target sequence, without requiring enzymatic cleavage.

This study describes the development of a novel HyTaq-LAMP assay capable of simultaneously detecting *M. pneumoniae* and distinguishing macrolide resistance-associated point mutations. The assay employs dual-labeled fluorescent probes that are structurally identical to conventional TaqMan probes, bearing a fluorophore at the 5′ end and a quencher at the 3′ end. However, unlike traditional TaqMan probes that require enzymatic cleavage for fluorescence generation, HyTaq probes emit fluorescence solely upon specific hybridization to their complementary DNA targets. This hybridization physically separates the fluorophore from the quencher, restoring fluorescence and enabling real-time signal generation without the need for 5′→3′ exonuclease activity (Fig. 1).

### Primer and probe design

Target regions within the *M. pneumoniae* 23S rRNA gene were selected based on known macrolide resistance-associated mutations at positions A2063G and A2064G. LAMP primers were designed using PrimerExplorer V5 (Eiken Chemical Co., Ltd., Tokyo, Japan) and manually refined to accommodate mutation site positioning and to ensure optimal primer performance. Next, three dual-labeled HyTaq probes were constructed: a FAM-labeled pan probe, a Cy5-labeled A2063G-specific probe, and a Texas Red-labeled A2064G-specific probe. All sequences used in the diagnostic assay are listed in Table S1. To investigate the fluorescence recovery mechanism of the HyTaq system, a separate set of probes (MP-P-21 through MP-P-5) was synthesized, all sharing the same nucleotide sequence as the MP pan probe but differing in F–Q configurations (Table 1). MP-P-21 was labeled with Cy5 at the 5′ end and BHQ2 at the 3′ end, while MP-P-17 through MP-P-5 were internally labeled with Cy5 and terminated with BHQ2. Additionally, MP-P-B and MP-P-C were designed to assess the influence of secondary structure. Complementary oligonucleotides (cOligos) were synthesized for each probe. All primers, probes, and cOligos were synthesized by Macrogen Inc. (Seoul, South Korea). Sequence information and labeling positions for probes used in mechanistic studies are provided in Table 1.

**TABLE 1:** MP-R HyTaq probes used in this study

Name	Sequence (5′–3′)
MP-P-21	[Cy5]-ACG GGG TCT TTC CGT CCC GTT-[BHQ2]
MP-P-17	ACG [Cy5 dG]GG TCT TTC CGT CCC GTT-[BHQ2]
MP-P-14	ACG GGG [Cy5 dT]CT TTC CGT CCC GTT-[BHQ2]
MP-P-11	ACG GGG TCT [Cy5 dT]TC CGT CCC GTT-[BHQ2]
MP-P-8	ACG GGG TCT TTC [Cy5 dC]GT CCC GTT-[BHQ2]
MP-P-5	ACG GGG TCT TTC CGT [Cy5 dC]CC GTT-[BHQ2]
MP-P-B	[Cy5]-CCG TTG CGC CTA ACG GGT GTC-[BHQ2]
MP-P-C	[Cy5]-CCC TTG GTT GTG TGC TGT TCT-[BHQ2]
MP-P complement oligo	TAG GCG CAA CGG GAC GGA AAG ACC CCG TGA AGC T
MP-P-B complement oligo	GTG AAG ACA CCC GTT AGG CGC AAC GGG ACG G
MP-P-C complement oligo	CAA TTA GAA CAG CAC ACA ACC AAG GGT AGT A
Non-complement oligo	CTG AAC CCC AAG GCC AAC CGG CTG GGG TGT TGA AGG TC

### Melting peak analysis

Each probe (final concentration: 0.5 µM) was mixed with an equimolar amount of its complementary oligonucleotide in a reaction mixture containing 2.5 µL of 10× isothermal amplification buffer (New England Biolabs, USA) and 1.5 µL of 100 mM MgSO_4_. Reactions were prepared in a total volume of 25 µL on ice and heated from 4°C to 95°C at a ramp rate of 1°C/30 seconds using a CFX96 real-time PCR system (Bio-Rad, USA). Fluorescence signals (Cy5 channels) were continuously monitored at 30 second intervals during the temperature increase, and melting behavior was assessed by tracking changes in fluorescence intensity as a function of temperature.

### Secondary structure prediction

To assess the presence and stability of probe secondary structures, *in silico* folding analysis was performed using NUPACK (http://www.nupack.org). Default parameters were used with a target temperature of 65°C and ionic conditions set to 50 mM Na^+^ and 2 mM Mg²^+^. Both minimum free energy (MFE) (ΔG) and ensemble free energy values were calculated for each probe.

### HyTaq-LAMP assay

The MP LAMP primer mix consisted of 4 µM each of outer primers (F3 and B3), 32 µM each of inner primers (FIP and BIP), and 10 µM each of loop primers (LF and LB). The probe mix contained 10 µM pan probe (FAM-labeled), 5 µM A2063G-specific probe (Cy5-labeled), and 5 µM A2064G-specific probe (Texas Red-labeled) (Table 1). The 25 µL reaction mixture included 12.5 µL of 2× LAMP Probe Master Mix (Elpis Biotech, South Korea), 1 µL of the primer mix, 1 µL of the probe mix, and 3 µL of extracted DNA template. Reactions were carried out in opaque tubes at 65°C for 40 minutes. Fluorescence signals were monitored in real time at 1 minute intervals using the CFX96 Real-Time PCR Detection System (Bio-Rad Laboratories, Hercules, CA, USA). Interpretation of results was based on probe-specific signal patterns: detection of FAM only indicated WT *M. pneumoniae*; simultaneous detection of FAM and Cy5 indicated the A2063G mutation; and detection of FAM and Texas Red indicated the A2064G mutation.

To evaluate whether probe-intrinsic secondary structure influences fluorescence recovery, the MP LAMP assay was performed using three probes (MP-P-21, MP-P-B, and MP-P-C) that differ in their predicted hairpin-forming propensities, as determined by NUPACK (http://www.nupack.org). Each 25 µL LAMP reaction contained 12.5 µL of 2× LAMP Probe Master Mix (Elpis Biotech, South Korea), primers at final concentrations of 4 µM (outer), 32 µM (inner), and 10 µM (loop), and 0.5 µM of the respective probe. Reactions were incubated at 65°C for 40 minutes in opaque tubes, and fluorescence was recorded in real time at 1 minute intervals using the CFX96 Real-Time PCR Detection System (Bio-Rad, USA).

### Allplex PneumoBacter Assay

The clinical performance of the MP-R HyTaq-LAMP assay was compared with that of the Allplex PneumoBacter Assay (Seegene Inc., South Korea), a commercially available multiplex real-time PCR assay. The assay was performed on a CFX96 Real-Time PCR Detection System (Bio-Rad Laboratories, Hercules, CA, USA) using 8 µL of extracted nucleic acid and 17 µL of PCR master mix (total volume: 25 µL), in accordance with the manufacturer’s instructions. Thermal cycling conditions consisted of incubation at 50°C for 20 minutes and 95°C for 15 minutes, followed by 45 cycles of 95°C for 10 seconds, 60°C for 1 minute, and 72°C for 10 seconds. Fluorescence signals were measured at both 60°C and 72°C during each cycle. According to the manufacturer’s guidelines, samples with Ct values ≤42 were interpreted as positive, whereas those with Ct values >42 or without amplification were considered negative. Runs in which both the target and internal control failed to produce signals were regarded as invalid and were repeated.

### Confirmation of genetic mutations by DNA sequencing

To confirm the presence of macrolide resistance-associated point mutations and evaluate the diagnostic accuracy of the MP-R HyTaq-LAMP assay, direct DNA sequencing was performed on all clinical samples. Real-time PCR was conducted in a 20 µL reaction volume using the EzAmp qPCR 2× Master Mix (Elpis Biotech, South Korea). The sequencing primer mix consisted of 10 µM each of the outer primers F3 (5′-TCT CTT GAC TGT CTC GGC T-3′) and B3 (5′-CCG TTA CCT TTT AGG AGG CG-3′), targeting the 23S rRNA gene region containing the A2063G and A2064G mutation sites. Each PCR reaction contained 10 µL of 2× master mix, 1 µL of the sequencing primer mix, 2 µL of the DNA sample, and 7 µL of RNase-free water. Amplification was carried out using the CFX96 Real-Time PCR Detection System (Bio-Rad Laboratories, Hercules, CA, USA) under the following cycling conditions: initial denaturation at 95°C for 10 minutes, followed by 45 cycles of 95°C for 30 seconds and 58°C for 40 seconds. The amplified PCR products were submitted to Macrogen Inc. (Seoul, South Korea) for Sanger sequencing. Sequencing results were used as the reference standard to assess the clinical sensitivity and specificity of the MP-R HyTaq-LAMP assay.

### Reproducibility test

The reproducibility of the MP-R HyTaq-LAMP assay was evaluated using *M. pneumoniae* WT, A2063G, and A2064G plasmids at three template concentrations: high (1 × 10⁷ copies/mL), medium (1 × 10⁵ copies/mL), and low (1 × 10³ copies/mL). Three independent operators each performed three replicate reactions per concentration on separate days to assess both intra-assay and inter-assay variability (*n* = 9 per concentration). For intra-assay reproducibility, the mean time-to-positive (Tp), standard deviation (SD), and coefficient of variation (%CV = SD/mean × 100) were calculated from each operator’s triplicate results. For inter-assay reproducibility, data from all three operators were combined, and overall mean, SD, and %CV values were computed across nine replicates. Amplification curves were monitored in real time using a fluorescence detection platform, and reactions showing no amplification within 40 minutes were considered negative.

### Analytical sensitivity determination

The analytical sensitivity of the MP-R HyTaq-LAMP assay was evaluated using both recombinant plasmid constructs and clinical specimens. For plasmid-based testing, plasmids containing partial sequences of the *M. pneumoniae* 23S rRNA gene representing the WT, A2063G, and A2064G mutations were synthesized by Macrogen Inc. (Seoul, South Korea) and cloned into the pMG-AMP vector at an initial concentration of 1 × 10⁹ copies/µL. Tenfold serial dilutions ranging from 10⁷ to 10^0^ copies/µL were prepared in nuclease-free water, along with a no-template control (NTC, water only). Each dilution was tested in triplicate using the MP-R HyTaq-LAMP assay. For clinical evaluation, NP swab specimens confirmed as either WT or A2063G mutant by Sanger sequencing (Macrogen Inc.) were serially diluted 10-fold for five steps using nuclease-free water, starting from the undiluted sample. Each dilution was tested in triplicate using both the MP-R HyTaq-LAMP assay and the commercial Allplex PneumoBacter Assay (Seegene Inc., South Korea) to compare detection sensitivity. The analytical sensitivity for both plasmid and clinical samples was defined as the lowest dilution at which all three replicates produced positive amplification.

## RESULTS

### Hybridization-dependent fluorescence recovery of dual-labeled ssDNA (HyTaq) probe

**Fig 2:**
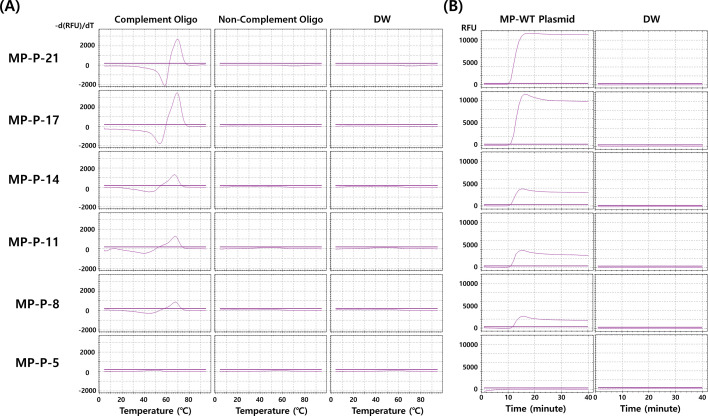
Evaluation of dual-labeled ssDNA probes with varying F–Q distances using melting peak analysis and LAMP assay. (**A**) Melting peak analysis was performed using six HyTaq probes (MP-P-21, MP-P-17, MP-P-14, MP-P-11, MP-P-8, and MP-P-5) in the presence of complementary or non-complementary oligonucleotides, or distilled water (DW). (**B**) LAMP assay using the same probes with MP-WT plasmid or DW. Fluorescence was recorded in real time at 1 minute intervals at 65°C.

**Fig 3:**
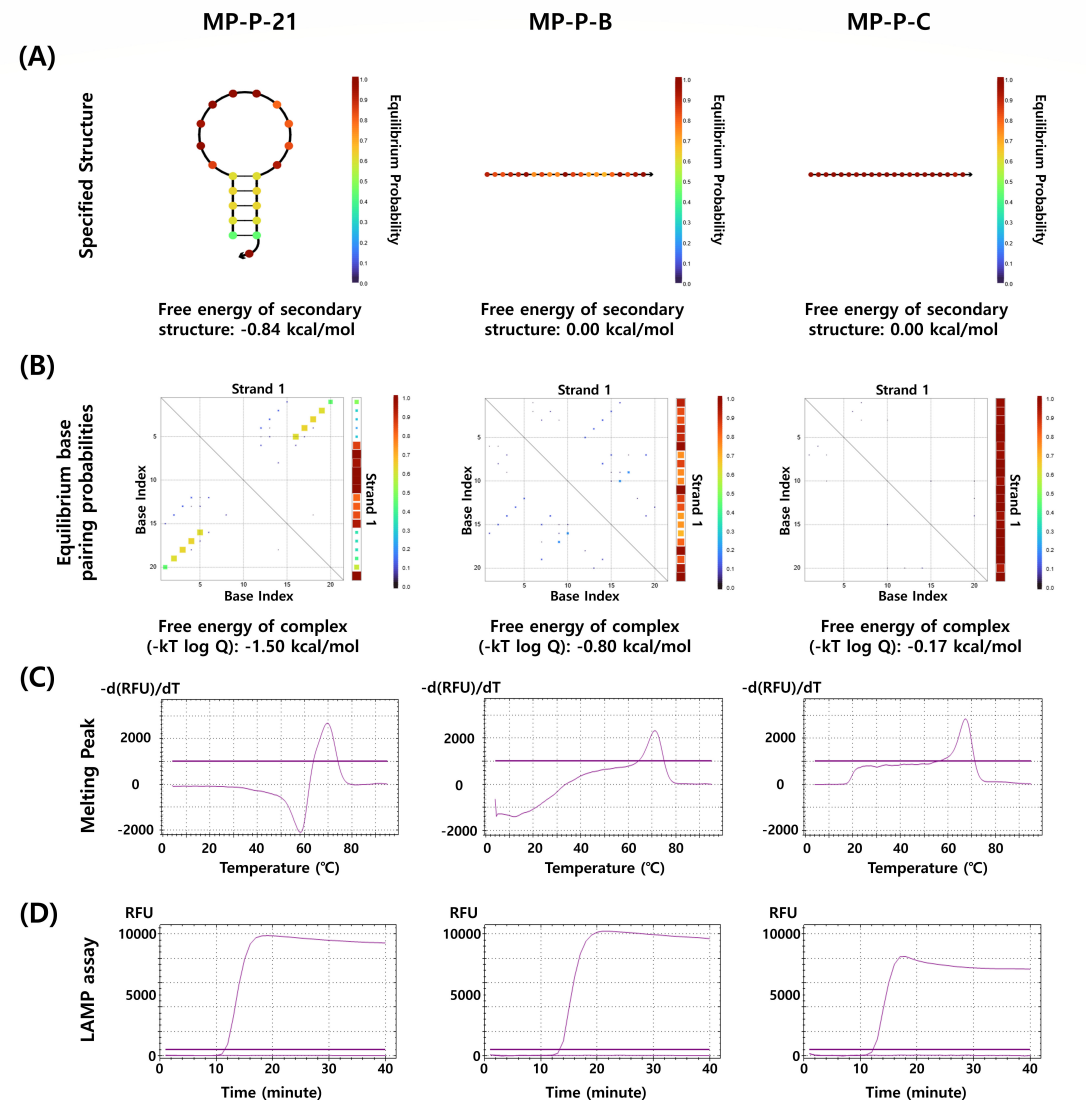
Structural and functional evaluation of three dual-labeled ssDNA probes in the HyTaq system. (**A**) Predicted secondary structures and MFE values for MP-P-21, MP-P-B, and MP-P-C as determined by NUPACK. (**B**) Equilibrium base-pairing probability matrices and corresponding ensemble free energies at 65°C for each probe. (**C**) Melting peak analysis of the three probes in the presence of complementary target sequences. (**D**) Real-time fluorescence profiles from LAMP assays using each probe with complementary targets, measured over 40 minutes at 65°C.

To investigate the mechanism of fluorescence recovery in the HyTaq system, we synthesized a panel of dual-labeled ssDNA probes with varying F–Q distances (21, 17, 14, 11, 8, and 5 nucleotides) and evaluated them using melting curve analysis, isothermal amplification, and *in silico* structure prediction. In melting peak analysis, MP-P-21 and MP-P-17 generated strong hybridization-dependent fluorescence with clear melting transitions, whereas probes with shorter F–Q distances (≤14 nt) showed progressively weaker or no signals. Importantly, no fluorescence was detected in mismatched or NTCs, confirming that signal generation was specific to probe–target hybridization (Fig. 2A). In isothermal LAMP assays, probes with F–Q distances ≥17 nt (MP-P-21 and MP-P-17) produced strong amplification signals, whereas shorter probes (≤14 nt) showed weaker or no signals. No fluorescence was observed in NTCs, confirming specificity. These results indicate that effective fluorescence recovery requires a minimum F–Q spacing of ~17 nucleotides, as shorter distances do not allow sufficient separation between fluorophore and quencher even upon hybridization (Fig. 2B). Next, to assess whether fluorescence recovery could result from disruption of secondary structures, we analyzed representative probes with (MP-P-21) and without (MP-P-B and MP-P-C) predicted hairpins using NUPACK. Despite differences in secondary structure stability, all probes generated strong hybridization-dependent fluorescence signals in both melting peak and LAMP assays (Fig. 3). These results indicate that fluorescence recovery in the HyTaq system is driven primarily by F–Q separation upon target hybridization rather than hairpin unfolding.

### Optimization of the MP-R HyTaq-LAMP assay

The MP-R HyTaq-LAMP assay is a multiplex isothermal amplification method designed to simultaneously detect *M. pneumoniae* and discriminate macrolide resistance-associated point mutations (A2063G and A2064G) within the 23S rRNA gene. In this assay, specimens showing only PAN positivity with negative A2063G and A2064G signals were interpreted as *M. pneumoniae* WT or potentially harboring resistance mutations outside of positions 2063 and 2064. By contrast, A2063G mutants yielded signals in both the PAN and Cy5 channels, while A2064G mutants were identified by concurrent positivity in the PAN and Texas Red channels. To establish optimal assay conditions, different probe ratios (pan:A2063G:A2064G = 10:5:5, 10:10:10, and 5:10:10 µM) and reaction temperatures (55°C, 60°C, and 65°C) were tested using plasmid templates (Fig. S1; Tables S2 and S3). Among the ratios, 10:5:5 provided the most efficient amplification, yielding earlier Ct values and stronger fluorescence for WT as well as both A2063G and A2064G mutants. Temperature testing showed that 65°C resulted in the fastest and strongest amplification without non-specific signals, whereas 60°C yielded slightly delayed and weaker signals and 55°C markedly reduced detection efficiency, particularly for the A2064G target. Based on these results, the 10:5:5 probe ratio and 65°C were selected as the optimal conditions for the MP-R HyTaq-LAMP assay.

### Analytical sensitivity of the MP-R HyTaq-LAMP assay using plasmid standards and clinical samples

The analytical sensitivity of the MP-R HyTaq-LAMP assay was evaluated using 10-fold serial dilutions (10⁷ to 10^0^ copies/µL) of recombinant plasmids containing *M. pneumoniae* 23S rRNA gene fragments representing the WT, A2063G, and A2064G targets. All targets were consistently detected down to 1 × 10³ copies/µL. The pan probe reacted with all plasmid templates as expected, whereas the A2063G and A2064G probes exhibited high specificity, detecting only their respective mutant sequences without cross-reactivity (Table 2). To assess clinical performance, NP swab specimens confirmed as either WT or A2063G mutant were serially diluted and tested in parallel using both the MP-R HyTaq-LAMP assay and the commercial Allplex PneumoBacter Assay (Seegene Inc., South Korea) (Table 3). In the WT sample, the MP-R HyTaq-LAMP assay detected down to the 10¹ dilution, while the Allplex assay detected down to 10², indicating one-log lower analytical sensitivity for the MP-R HyTaq-LAMP assay. For the A2063G mutant, both assays demonstrated comparable sensitivity, detecting down to 10². No cross-reactivity was observed in any non-target probe channels, confirming the high specificity of the MP-R HyTaq-LAMP assay in clinical samples. All plasmid-based and clinical dilution experiments were conducted in triplicate, and the analytical sensitivity was determined as the lowest dilution at which all three replicates yielded positive results. These findings indicate that although the MP-R HyTaq-LAMP assay showed slightly lower analytical sensitivity for WT detection compared with the reference PCR assay, it maintained reliable clinical performance with the added advantages of faster turnaround and simplified isothermal operation.

**TABLE 2:** Analytical sensitivity of the MP-R HyTaq-LAMP assay for *M. pneumoniae* WT, A2063G, and A2064G plasmid targets^*a*^

Copies/μL	MP-R HyTaq-LAMP assay								
	MP-WT plasmid (Tp ± SD)			MP-A2063G plasmid (Tp ± SD)			MP-A2064G plasmid (Tp ± SD)		
	Pan	A2063G	A2064G	Pan	A2063G	A2064G	Pan	A2063G	A2064G
1 × 10^7^	11.58 ± 0.12	ND	ND	12.14 ± 0.03	12.61 ± 0.06	ND	11.45 ± 0.03	ND	12.16 ± 0.02
1 × 10^6^	12.84 ± 0.17	ND	ND	13.13 ± 0.03	13.57 ± 0.04	ND	12.84 ± 0.13	ND	13.46 ± 0.10
1 × 10^5^	14.30 ± 0.13	ND	ND	14.25 ± 0.18	14.75 ± 0.29	ND	14.18 ± 0.14	ND	14.78 ± 0.20
1 × 10^4^	15.75 ± 1.70	ND	ND	16.90 ± 0.72	17.36 ± 0.68	ND	17.13 ± 0.64	ND	17.52 ± 0.56
1 × 10^3^	21.20 ± 9.29	ND	ND	23.62 ± 2.86	23.26 ± 2.98	ND	19.90 ± 2.38	ND	20.23 ± 2.21
1 × 10^2^	ND	ND	ND	ND	ND	ND	ND	ND	ND
1 × 10^1^	ND	ND	ND	ND	ND	ND	ND	ND	ND
1 × 10^0^	ND	ND	ND	ND	ND	ND	ND	ND	ND
DW	ND	ND	ND	ND	ND	ND	ND	ND	ND

^*a*^ Tp, time to positive; ND, not detected.

**TABLE 3:** Comparative analytical sensitivity of MP-R HyTaq-LAMP and Allplex assays using diluted clinical specimens of *M. pneumoniae* WT and A2063G mutant^*a*^

Dilution	*M. pneumoniae* WT				*M. pneumoniae* A2063G mutant			
	Allplex assay (Ct ± SD)	MP-R HyTaq-LAMP assay (Tp ± SD)			Allplex assay (Ct ± SD)	MP-R HyTaq-LAMP assay (Tp ± SD)		
		Pan	A2063G	A2064G		Pan	A2063G	A2064G
10^0^	32.02 ± 0.18	15.05 ± 0.04	ND	ND	31.45 ± 0.05	13.71 ± 0.35	13.61 ± 0.34	ND
10^1^	35.09 ± 0.14	20.43 ± 0.76	ND	ND	34.60 ± 0.15	15.63 ± 0.58	15.49 ± 0.42	ND
10^2^	39.24 ± 1.51	ND	ND	ND	39.81 ± 0.55	22.86 ± 1.97	20.41 ± 2.25	ND
10^3^	ND	ND	ND	ND	ND	ND	ND	ND
10^4^	ND	ND	ND	ND	ND	ND	ND	ND
10^5^	ND	ND	ND	ND	ND	ND	ND	ND
DW	ND	ND	ND	ND	ND	ND	ND	ND

^*a*^ Ct, cycle threshold; Tp, time to positive; ND, not detected.

### Clinical performance of the MP-R HyTaq-LAMP assay in comparison with the Allplex PneumoBacter Assay

The clinical performance of the MP-R HyTaq-LAMP assay was evaluated using 224 NP swab specimens, including 103 positive and 121 negative samples. All positive samples were confirmed by sequencing and categorized as either WT or carrying the A2063G mutation in the 23S rRNA gene. Eight samples were WT, and 95 were A2063G mutants. No samples harboring the A2064G mutation were identified among the clinical specimens (Table 4). For WT detection, both the MP-R HyTaq-LAMP assay and the Allplex assay identified all eight samples, showing 100% sensitivity. For A2063G detection, the MP-R HyTaq-LAMP assay detected 91 out of 95 positive samples using both the pan and A2063G probe channels, corresponding to a sensitivity of 95.79%. In contrast, the Allplex assay detected all 95 samples, achieving 100% sensitivity. The MP-R HyTaq-LAMP assay demonstrated an overall clinical sensitivity of 96.12% (99/103), slightly lower than that of the Allplex assay, which showed 100% (103/103). For the 121 negative samples, both assays showed perfect agreement, yielding 100% specificity with no false-positive or non-specific signals observed. Notably, all positive results from the MP-R HyTaq-LAMP assay were accurately assigned to either the WT or A2063G category, with no misclassification or cross-reactivity observed between genotypes. These results indicate that the MP-R HyTaq-LAMP assay can reliably differentiate *M. pneumoniae* WT and A2063G mutant strains with high specificity and clinical accuracy.

**TABLE 4:** Comparison of clinical sensitivity and specificity of the MP-R HyTaq-LAMP assay and Allplex PneumoBacter Assay^*a*^

Clinical samples		Allplex PneumoBacter Assay	MP-R HyTaq-LAMP assay		
			Pan	A2063G	A2064G
*M. pneumoniae* WT (*n* = 8)	P/total	8/8	8/8	0/8	0/8
	Sensitivity (95% CI)	100% (63.06–100)	100% (63.06–100)	–	–
	Specificity (95% CI)	–	–	100% (63.06–100)	100% (63.06–100)
*M. pneumoniae* A2063G (*n* = 95)	P/total	95/95	91/95	91/95	0/95
	Sensitivity (95% CI)	100% (96.18–100)	95.79% (89.57–98.84)	95.79% (89.57–98.84)	–
	Specificity (95% CI)	–	–	–	100% (96.18–100)
Non-infected (*n* = 121)	N/total	0/121	0/121	0/121	0/121
	Sensitivity (95% CI)	–	–	–	–
	Specificity (95% CI)	100% (97.00–100)	100% (97.00–100)	100% (97.00–100)	100% (97.00–100)

^*a*^ P, positive; N, negative; CI, confidence interval; –, not applicable.

### Reproducibility of the MP-R HyTaq-LAMP assay

The reproducibility of the MP-R HyTaq-LAMP assay was assessed using *M. pneumoniae* WT, A2063G, and A2064G plasmids at high (1 × 10⁷), medium (1 × 10⁵), and low (1 × 10³ copies/µL) concentrations. Three independent operators each performed three replicate reactions per concentration (*n* = 9). All replicates consistently yielded amplification curves with %CV values below 5% for medium-to-high copy numbers, confirming robust intra-assay and inter-assay precision (Tables 5 and 6). At the lowest concentration (10³ copies/µL), amplification was still reproducible though slightly variable across operators, which is expected near the assay’s analytical detection limit. No false-positive reactions were observed in any run.

**TABLE 5:** Intra-assay reproducibility of the MP-R HyTaq-LAMP assay

Sample	Copies/µL	MP-R HyTaq-LAMP assay	Intra-assay (mean ± SD [%CV])		
			Operator 1 (*n* = 3)	Operator 2 (*n* = 3)	Operator 3 (*n* = 3)
MP-WT plasmid	10^7^	PAN	11.58 ± 0.12 (1.01)	11.41 ± 0.09 (0.81)	11.36 ± 0.18 (1.60)
		A2063G	ND	ND	ND
		A2064G	ND	ND	ND
	10^5^	PAN	14.30 ± 0.13 (0.91)	14.28 ± 0.32 (2.26)	14.32 ± 0.38 (2.67)
		A2063G	ND	ND	ND
		A2064G	ND	ND	ND
	10^3^	PAN	21.20 ± 9.29 (43.82)	21.69 ± 0.98 (4.50)	20.87 ± 2.71 (12.99)
		A2063G	ND	ND	ND
		A2064G	ND	ND	ND
MP-A2063G plasmid	10^7^	PAN	12.14 ± 0.03 (0.25)	12.30 ± 0.14 (1.10)	12.23 ± 0.06 (0.49)
		A2063G	12.61 ± 0.06 (0.44)	13.26 ± 0.15 (1.13)	13.20 ± 0.10 (0.72)
		A2064G	ND	ND	ND
	10^5^	PAN	14.25 ± 0.18 (1.30)	14.47 ± 0.37 (2.57)	14.55 ± 0.36 (2.49)
		A2063G	14.75 ± 0.29 (2.00)	15.65 ± 0.20 (1.28)	15.51 ± 0.39 (2.52)
		A2064G	ND	ND	ND
	10^3^	PAN	23.35 ± 2.86 (12.23)	21.62 ± 0.90 (4.15)	21.49 ± 1.20 (5.59)
		A2063G	23.26 ± 2.95 (12.68)	23.06 ± 0.91 (3.95)	23.05 ± 1.40 (6.09)
		A2064G	ND	ND	ND
MP-A2064G plasmid	10^7^	PAN	11.45 ± 0.03 (0.27)	11.32 ± 0.16 (1.40)	11.48 ± 0.05 (0.40)
		A2063G	ND	ND	ND
		A2064G	12.16 ± 0.02 (0.17)	12.39 ± 0.20 (1.61)	12.56 ± 0.03 (0.28)
	10^5^	PAN	14.18 ± 0.14 (0.98)	14.68 ± 0.59 (4.03)	14.79 ± 0.67 (4.53)
		A2063G	ND	ND	ND
		A2064G	14.78 ± 0.20 (1.34)	16.15 ± 0.03 (0.19)	15.94 ± 0.73 (4.58)
	10^3^	PAN	22.12 ± 4.19 (18.95)	20.78 ± 0.37 (1.79)	20.26 ± 1.90 (9.36)
		A2063G	ND	ND	ND
		A2064G	21.52 ± 2.73 (12.66)	23.06 ± 1.30 (5.63)	22.00 ± 2.33 (10.58)

**TABLE 6:** Inter-assay reproducibility of the MP-R HyTaq-LAMP assay

Sample	Copies/µL	Inter-assay (three operators, each tested in triplicate)								
		MP-R HyTaq-LAMP assay								
		PAN			A2063G			A2064G		
		Mean	SD	%CV	Mean	SD	%CV	Mean	SD	%CV
MP-WT plasmid	1 × 10^7^	11.45	0.12	1.04	ND	ND	ND	ND	ND	ND
	1 × 10^5^	14.30	0.02	0.15	ND	ND	ND	ND	ND	ND
	1 × 10^3^	21.25	0.41	1.94	ND	ND	ND	ND	ND	ND
MP-A2063G plasmid	1 × 10^7^	12.23	0.08	0.67	13.02	0.36	2.74	ND	ND	ND
	1 × 10^5^	14.42	0.16	1.10	15.31	0.49	3.18	ND	ND	ND
	1 × 10^3^	22.15	1.04	4.69	23.12	0.12	0.52	ND	ND	ND
MP-A2064G plasmid	1 × 10^7^	11.42	0.08	0.72	ND	ND	ND	12.37	0.20	1.61
	1 × 10^5^	14.55	0.33	2.25	ND	ND	ND	15.62	0.73	4.70
	1 × 10^3^	21.05	0.96	4.56	ND	ND	ND	22.19	0.79	3.56

### Cross-reactivity testing of the MP-R HyTaq-LAMP assay

The cross-reactivity of the MP-R HyTaq-LAMP assay was assessed using a panel of 16 clinically relevant respiratory pathogens, including *Bordetella pertussis*, *Candida albicans*, *Escherichia coli*, *Klebsiella pneumoniae*, *Pseudomonas aeruginosa*, *Staphylococcus aureus*, *Staphylococcus epidermidis*, *Streptococcus agalactiae*, *Streptococcus pneumoniae*, *Streptococcus pyogenes*, human parainfluenza virus types 1–4, human rhinovirus, and RSV (Table S4). No amplification signals were detected in any of the target channels (Pan, A2063G, and A2064G), indicating a lack of cross-reactivity with non-target organisms. These results suggest that the MP-R HyTaq-LAMP assay has high specificity for *M. pneumoniae* under the tested conditions.

## DISCUSSION

The growing prevalence of MRMP poses a serious threat to the effective management of community-acquired respiratory infections, particularly among pediatric populations in Asia. Previous studies have consistently reported that the A2063G mutation in domain V of the 23S rRNA gene is the predominant mechanism underlying macrolide resistance, with the A2064G mutation also observed in regions such as China and Japan (31, 32). Consequently, rapid and accurate molecular diagnostic tools capable of simultaneously detecting *M. pneumoniae* and identifying resistance-associated mutations are critically needed to inform timely and effective antimicrobial interventions (33).

In this study, we developed and validated the MP-R HyTaq-LAMP assay, a novel isothermal multiplex assay that enables simultaneous detection of *M. pneumoniae* and discrimination of macrolide resistance mutations (A2063G and A2064G) in a single-tube reaction. The assay’s key innovation lies in its use of dual-labeled TaqMan-style probes that function under standard Bst-based LAMP conditions without requiring 5′→3′ exonuclease activity. Traditionally, TaqMan probes generate fluorescence upon hybridization to the target sequence, followed by enzymatic cleavage of the probe by Taq polymerase, which releases the fluorophore from the quencher. However, since Bst polymerase lacks 5′→3′ exonuclease activity, this cleavage-dependent mechanism has not been applicable to standard LAMP reactions to date (34, 35). Recently, Liang et al. demonstrated a modified approach in which conventional TaqMan probes were successfully used in LAMP by employing Bst 5.0 polymerase, which possesses 5′→3′ exonuclease activity, thereby enabling fluorescence generation via the canonical cleavage mechanism (22). In contrast, the HyTaq system presented here operates with standard Bst polymerase, which lacks such exonuclease activity. Remarkably, structurally identical dual-labeled probes still produced strong signals under these exonuclease-deficient conditions, operating instead through a hybridization-driven quenching reversal mechanism. In this mechanism, fluorescence is restored solely by the physical separation of the fluorophore and quencher upon target-specific duplex formation, independent of enzymatic cleavage. This mechanism was substantiated through melting curve analysis, real-time isothermal fluorescence detection, and *in silico* secondary structure modeling, collectively supporting the proposed hybridization-driven mechanism. Importantly, probes lacking stable secondary structures also generated signal, confirming that HyTaq differs fundamentally from molecular beacons, which rely on hairpin unfolding for signal generation. To further investigate this mechanism, we systematically tested a series of probes with varying F–Q distances. Probes with F–Q separations of ≥17 nucleotides produced robust hybridization-dependent fluorescence, while those with ≤14 nucleotides failed to generate sufficient signal despite full complementarity, suggesting a spatial threshold for effective quenching reversal upon duplex formation. Although these results strongly support a hybridization-driven quenching reversal mechanism, we cannot completely exclude the possibility that subtle conformational effects of the probe or template may also contribute to fluorescence recovery. Future biophysical studies will be valuable to clarify whether such auxiliary mechanisms play a role.

The MP-R HyTaq-LAMP assay demonstrated excellent analytical performance, with a limit of detection of 1 × 10³ copies/μL for WT, A2063G, and A2064G plasmid templates. Clinical validation using 224 NP specimens demonstrated high sensitivity and specificity, with sensitivities of 100% for WT and 95.79% for A2063G-positive samples, and 100% specificity. The A2064G mutation was not detected in clinical samples, consistent with its low regional prevalence (36), but was specifically detected using synthetic templates. Although the MP-R HyTaq-LAMP assay showed approximately one-log lower analytical sensitivity for WT detection compared with the Allplex assay, this difference did not translate into a major clinical impact. The assay successfully detected 96% (99/103) of Allplex assay-positive WT clinical samples. No cross-reactivity was observed with 16 common respiratory pathogens, supporting high analytical specificity.

One limitation of this study is the limited availability of clinical samples, including a relatively small number of WT cases (*n* = 8) and the absence of specimens harboring the A2064G mutation. These constraints may restrict the robustness of performance estimates for these subgroups but largely reflect local epidemiological patterns rather than technical shortcomings of the assay. Future multicenter studies in regions with higher prevalence of WT and A2064G strains will be important to further validate the multiplex design. Another limitation is that the analytical sensitivity evaluation was based on three replicates of 10-fold dilutions; a more precise determination would require additional replicates and finer bracketing dilutions, which will be addressed in future studies.

In summary, the MP-R HyTaq-LAMP assay provides a rapid, cost-effective, and multiplex-compatible molecular diagnostic solution for detecting *M. pneumoniae* and clinically relevant macrolide resistance mutations. By leveraging a novel hybridization-driven fluorescence mechanism under standard LAMP conditions, this platform eliminates the need for enzymatic cleavage, specialized equipment, or additional reaction components. Importantly, this mechanism is distinct from both cleavage-dependent TaqMan probes and hairpin-dependent molecular beacons, thereby expanding the scope of probe-based diagnostics to widely used isothermal platforms. These attributes render it particularly suitable for decentralized and point-of-care testing in both clinical and resource-limited settings.

## Data Availability

The raw data supporting the findings of this study have been deposited in Zenodo and are publicly available (DOI: 10.5281/zenodo.17694234).

## SUPPLEMENTAL MATERIAL

Supplemental material jcm.01291-25-s0001.docx Tables S1 to S6; Fig. S1.

ASM does not own the copyrights to Supplemental Material that may be linked to, or accessed through, an article. The authors have granted ASM a non-exclusive, world-wide license to publish the Supplemental Material files. Please contact the corresponding author directly for reuse.
