# Supplementary material for: Development of a LAMP assay using hybridization-based TaqMan-style probes (HyTaq) for simultaneous detection of wild-type and macrolide-resistant Mycoplasma pneumoniae
Source: J Clin Microbiol. 2025 Dec 17;64(1):e01291-25. doi: 10.1128/jcm.01291-25 (PMC12802244; doi:10.1128/jcm.01291-25)
Supplement: Supplemental material — Tables S1 to S6; Fig. S1. [file jcm.01291-25-s0001.docx]

**Table S1** MP-R HyTaq-LAMP primer sets used in this study.

| **Mixture** | **Target Gene** | **Name** | **Sequence (5’-3’)** | **μM** |
| --- | --- | --- | --- | --- |
| MP LAMP Primer Mix | 23S rRNA | MP F3 | TCT CTT GAC TGT CTC GGC T | 4 |
|  |  | MP B3 | CCG TTA CCT TTT AGG AGG CG | 4 |
|  |  | MP FIP | GCA TCG ATT GCT CCT ACC CTC GGT GAA ATC CAG GTA CG | 32 |
|  |  | MP BIP | GGA CTT GTT GAT GCG AAA GGA CTG CCC ACC TAA CAC TGT | 32 |
|  |  | MP LF | ACG GGG TCT TTC CGT CCC GTT | 10 |
|  |  | MP LB | TGG AAT ACT ACC CTT GGT TG T GTG C | 10 |
| HyTaq Probe Mix | 23S rRNA | MP pan P | [FAM]-CCG TTG CGC CTA ACG GGT GTC-[BHQ1] | 10 |
|  |  | A2063G P | [Cy5]-ACG GGG TCT TCC CGT CCC GTT-[BHQ2] | 5 |
|  |  | A2064G P | [Texas Red]-ACG GGG TCT CTC CGT CCC GTT-[BHQ1] | 5 |

**Table S2** Optimization of probe concentration ratios for the MP-R HyTaq-LAMP assay

| **pan:A2063G:A2064G probe ratio (µM)** | **1 × 10^6^ copies/µL** | **MP-R HyTaq-LAMP assay Ct Values** | | | | |
| --- | --- | --- | --- | --- | --- | --- |
|  |  | **pan** | | **A2063G** | | **A2064G** |
| 10:5:5 | MP-WT plasmid | 10.64 ± 0.23 | ND | | ND | |
|  | MP-A2063G plasmid | 12.04 ± 0.12 | 12.80 ± 0.21 | | ND | |
|  | MP-A2064G plasmid | 11.81 ± 0.29 | ND | | 13.08 ± 0.26 | |
| 10:10:10 | MP-WT plasmid | 10.62 ± 0.16 | ND | | ND | |
|  | MP-A2063G plasmid | 11.52 ± 0.27 | 12.83 ± 0.24 | | ND | |
|  | MP-A2064G plasmid | 11.39 ± 0.07 | ND | | 13.12 ± 0.28 | |
| 5: 10:10 | MP-WT plasmid | 10.66 ± 0.03 | ND | | ND | |
|  | MP-A2063G plasmid | 11.26 ± 0.08 | 11.60 ± 0.11 | | ND | |
|  | MP-A2064G plasmid | 11.17 ± 0.16 | ND | | 11.93 ± 0.25 | |

**Table S3** Temperature optimization of the MP-R HyTaq-LAMP Assay

| **Temp(ºC)** | **1 × 10^6^ copies/µL** | **MP-R HyTaq-LAMP assay Ct Values** | | |
| --- | --- | --- | --- | --- |
|  |  | **pan** | **A2063G** | **A2064G** |
| 65.0 ºC | MP-WT plasmid | 10.66 ± 0.03 | ND | ND |
|  | MP-A2063G plasmid | 11.26 ± 0.08 | 11.60 ± 0.11 | ND |
|  | MP-A2064G plasmid | 11.17 ± 0.16 | ND | 11.93 ± 0.25 |
| 60.0 ºC | MP-WT plasmid | 12.01 ± 0.15 | 14.16 ± 0.30 | ND |
|  | MP-A2063G plasmid | 12.42 ± 2.94 | 14.26 ± 0.20 | ND |
|  | MP-A2064G plasmid | 12.50 ± 0.22 | 15.30 ± 0.19 | 14.09 ± 0.13 |
| 55.0 ºC | MP-WT plasmid | 18.54 ± 0.35 | 20.22 ± 0.39 | ND |
|  | MP-A2063G plasmid | 20.50 ± 0.28 | 21.01 ± 0.26 | ND |
|  | MP-A2064G plasmid | 19.30 ± 0.57 | 20.96 ± 0.49 | 21.92 ± 0.55 |

**Table S4**. Cross-reactivity testing of the MP-R HyTaq-LAMP Assay with common respiratory pathogens.

| **Pathogens** | **Confirmation method / Reference** | **Ct values** | **MP-R HyTaq-LAMP assay** | | |
| --- | --- | --- | --- | --- | --- |
|  |  |  | **Pan** | **A2063G** | **A2064G** |
| *Bordetella pertusis* | Allplex™ PneumoBacter Assay (Seegene Inc., South Korea) | 32.15 | ND | ND | ND |
| *Streptococcus pneumoniae* |  | 32.24 | ND | ND | ND |
| Human parainfluenza virus type 1 | Allplex™ RV Master Assay (Seegene Inc., South Korea) | 28.91 | ND | ND | ND |
| Human parainfluenza virus type 2 |  | 31.05 | ND | ND | ND |
| Human parainfluenza virus type 3 |  | 30.6 | ND | ND | ND |
| Human parainfluenza virus type 4 |  | 24.94 | ND | ND | ND |
| Human rhinovirus |  | 25.04 | ND | ND | ND |
| Respiratory syncytial virus |  | 21.75 | ND | ND | ND |
| *Escherichia coli* | (23) | 15.06 | ND | ND | ND |
| *Straphylococcus aureus* | (24) | 18.22 | ND | ND | ND |
| *Streptococcus agalactiae* | (25) | 24.08 | ND | ND | ND |
| *Streptococcus pyogenes* | (26) | 24.83 | ND | ND | ND |
| *Candida albicans* | (27) | 23.57 | ND | ND | ND |
| *Klebsiella pneumoniae* | (28) | 25.39 | ND | ND | ND |
| *Straphylococcus epidermidis* | (29) | 20.9 | ND | ND | ND |
| *Pseudomonas aeruginosa* | (30) | 35.11 | ND | ND | ND |

**Table S5.** Intra-assay Reproducibility of the MP-R HyTaq-LAMP Assay

| Sample | copies/µL | MP-R HyTaq-LAMP Assay | Intra-assay (Mean±SD (%CV)) | | | | |
| --- | --- | --- | --- | --- | --- | --- | --- |
|  |  |  | Operator 1 (n=3) | Operator 2 (n=3) | | Operator 3 (n=3) | |
| MP wt plasmid | 10^7 | PAN | 11.58±0.12 (1.01) | | 11.41±0.09(0.81) | | 11.36±0.18 (1.60) |
|  |  | A2063G | ND | | ND | | ND |
|  |  | A2064G | ND | | ND | | ND |
|  | 10^5 | PAN | 14.30±0.13 (0.91) | | 14.28±0.32 (2.26) | | 14.32±0.38 (2.67) |
|  |  | A2063G | ND | | ND | | ND |
|  |  | A2064G | ND | | ND | | ND |
|  | 10^3 | PAN | 21.20±9.29 (43.82) | | 21.69±0.98 (4.50) | | 20.87±2.71 (12.99) |
|  |  | A2063G | ND | | ND | | ND |
|  |  | A2064G | ND | | ND | | ND |
| MP-A2063G plasmid | 10^7 | PAN | 12.14±0.03 (0.25) | | 12.30±0.14 (1.10) | | 12.23±0.06 (0.49) |
|  |  | A2063G | 12.61±0.06 (0.44) | | 13.26±0.15 (1.13) | | 13.20±0.10 (0.72) |
|  |  | A2064G | ND | | ND | | ND |
|  | 10^5 | PAN | 14.25±0.18 (1.30) | | 14.47±0.37 (2.57) | | 14.55±0.36 (2.49) |
|  |  | A2063G | 14.75±0.29 (2.00) | | 15.65±0.20 (1.28) | | 15.51±0.39 (2.52) |
|  |  | A2064G | ND | | ND | | ND |
|  | 10^3 | PAN | 23.35±2.86 (12.23) | | 21.62±0.90 (4.15) | | 21.49±1.20 (5.59) |
|  |  | A2063G | 23.26±2.95 (12.68) | | 23.06±0.91 (3.95) | | 23.05±1.40 (6.09) |
|  |  | A2064G | ND | | ND | | ND |
| MP-A2064G plasmid | 10^7 | PAN | 11.45±0.03 (0.27) | | 11.32±0.16 (1.40) | | 11.48±0.05 (0.40) |
|  |  | A2063G | ND | | ND | | ND |
|  |  | A2064G | 12.16±0.02 (0.17) | | 12.39±0.20 (1.61) | | 12.56±0.03 (0.28) |
|  | 10^5 | PAN | 14.18±0.14 (0.98) | | 14.68±0.59 (4.03) | | 14.79±0.67 (4.53) |
|  |  | A2063G | ND | | ND | | ND |
|  |  | A2064G | 14.78±0.20 (1.34) | | 16.15±0.03 (0.19) | | 15.94±0.73 (4.58) |
|  | 10^3 | PAN | 22.12±4.19 (18.95) | | 20.78±0.37 (1.79) | | 20.26±1.90 (9.36) |
|  |  | A2063G | ND | | ND | | ND |
|  |  | A2064G | 21.52±2.73 (12.66) | | 23.06±1.30 (5.63) | | 22.00±2.33 (10.58) |

**Table S6.** Inter-assay Reproducibility of the MP-R HyTaq-LAMP Assay

| Sample | copies/µL | Inter-assay (Three operators, each tested in triplicate) | | | | | | | | |
| --- | --- | --- | --- | --- | --- | --- | --- | --- | --- | --- |
|  |  | MP-R HyTaq-LAMP Assay | | | | | | | | |
|  |  | PAN | | | 2063G | | | A2064G | | |
|  |  | Mean | SD | %CV | Mean | SD | %CV | Mean | SD | %CV |
| MP-WT plasmid | 1ⅹ10^7^ | 11.45 | 0.12 | 1.04 | ND | ND | ND | ND | ND | ND |
|  | 1ⅹ10^5^ | 14.30 | 0.02 | 0.15 | ND | ND | ND | ND | ND | ND |
|  | 1ⅹ10^3^ | 21.25 | 0.41 | 1.94 | ND | ND | ND | ND | ND | ND |
| MP-A2063G plasmid | 1ⅹ10^7^ | 12.23 | 0.08 | 0.67 | 13.02 | 0.36 | 2.74 | ND | ND | ND |
|  | 1ⅹ10^5^ | 14.42 | 0.16 | 1.10 | 15.31 | 0.49 | 3.18 | ND | ND | ND |
|  | 1ⅹ10^3^ | 22.15 | 1.04 | 4.69 | 23.12 | 0.12 | 0.52 | ND | ND | ND |
| MP-A2064G plasmid | 1ⅹ10^7^ | 11.42 | 0.08 | 0.72 | ND | ND | ND | 12.37 | 0.20 | 1.61 |
|  | 1ⅹ10^5^ | 14.55 | 0.33 | 2.25 | ND | ND | ND | 15.62 | 0.73 | 4.70 |
|  | 1ⅹ10^3^ | 21.05 | 0.96 | 4.56 | ND | ND | ND | 22.19 | 0.79 | 3.56 |


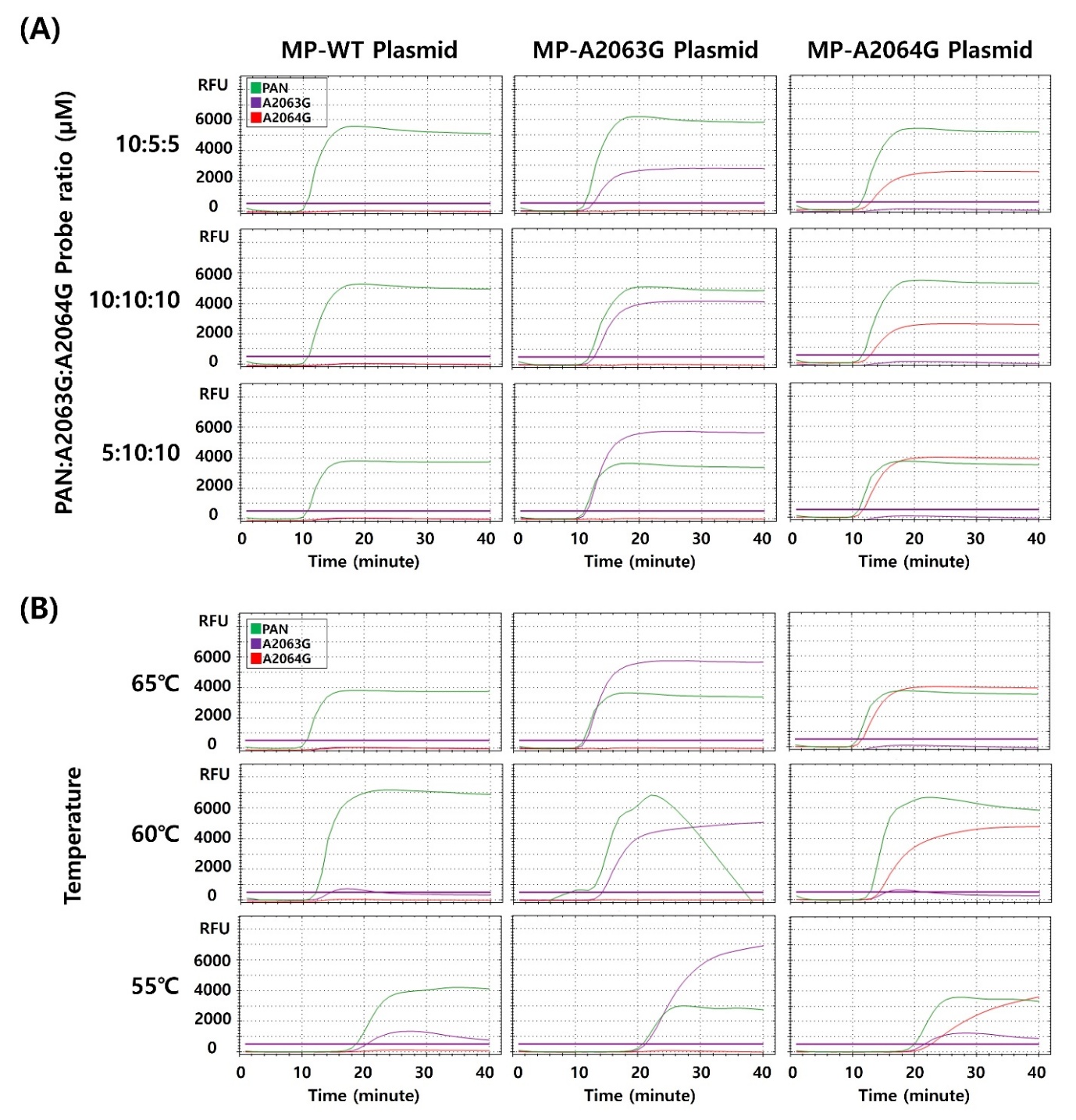


**Fig. S1 Optimization of probe ratio and reaction temperature for the MP-R HyTaq-LAMP assay.** (A) Evaluation of three probe ratios (pan:A2063G:A2064G = 10:5:5, 10:10:10, and 5:10:10 µM) using synthetic plasmids representing wild-type (WT), A2063G mutant, and A2064G mutant. Each panel shows the amplification curve for the corresponding target and probe ratio. (B) Temperature optimization of the MP-R HyTaq-LAMP assay at 65.0 °C, 60.0 °C, and 55.0 °C using the optimized probe ratio (10:5:5). Amplification curves were generated using synthetic plasmids (1 × 10⁶ copies/µL) for WT, A2063G, and A2064G.
